# Low hospital admission rates for respiratory diseases in children

**DOI:** 10.1186/1471-2296-11-76

**Published:** 2010-10-09

**Authors:** Johannes HJM Uijen, François G Schellevis, Patrick JE Bindels, Sten P Willemsen, Johannes C van der Wouden

**Affiliations:** 1 Department of General Practice, Erasmus MC University Medical Center, Rotterdam, the Netherlands; 2 NIVEL, Netherlands Institute for Health Services Research, Utrecht, the Netherlands; and Department of General Practice EMGO Institute, VU University Medical Center, Amsterdam, the Netherlands

## Abstract

**Background:**

Population-based data on hospital admissions for children aged 0-17 years concerning all respiratory diseases are scarce. This study examined hospital admissions in relation to the preceding consultations in general practice in this age group.

**Methods:**

Data on children aged 0-17 years with respiratory diseases included in the Second Dutch National Survey of General Practice (DNSGP-2) were linked to all hospital admissions in the Dutch National Medical Registration. Admission rates for respiratory diseases were calculated. Data were analysed using multivariate logistic regression.

**Results:**

Of all 79,272 children within the DNSGP-2, 1.8% were admitted to hospital for any respiratory diagnosis. The highest admission rates per 1000 children were for chronic disease of tonsils and adenoids (12.9); pneumonia and influenza (0.97); and asthma (0.92). Children aged 0-4 years and boys were admitted more frequently. Of children with asthma, 2.3% were admitted for respiratory diseases. For asthma, admission rates varied by urbanisation level: 0.47/1000 children/year in cities with ≤ 30,000 inhabitants, 1.12 for cities with ≥ 50,000 inhabitants, and 1.73 for the three largest cities (p = 0.002). Multivariate logistic regression showed that within two weeks after a GP consultation, younger age (OR 0.81, 95% CI 0.76-0.88) and more severe respiratory diseases (5.55, 95% CI 2.99-8.11) predicted hospital admission.

**Conclusions:**

Children in the general population with respiratory diseases (especially asthma) had very low hospital admission rates. In urban regions children were more frequently admitted due to respiratory morbidity. For effectiveness studies in a primary care setting, hospital admission rates should not be used as quality end-point.

## Background

Respiratory symptoms account for about 25% of consultations for children in general practice [1]. Respiratory tract infections are the leading cause of childhood hospital admission [2-4], which is often an unpleasant experience for the child and their parents. Data on hospital admissions for children aged 0-17 years concerning the total spectrum of respiratory diseases are scarce. Generally, data on a specific age group (e.g. preschool children) or on a specific respiratory disease (e.g. asthma) are reported [5-7]. Most admission data are hospital based [8], but the catchment area and hence the denominator for calculating these hospital admission rates is often not known. Primary care data linked with hospital admission registration data can be useful for allocating resources, to plan hospital care, to predict admissions for children from general practice, and studies conducted in primary and specialised care might benefit from these data.

In recent decades various factors have influenced morbidity patterns in children with respiratory diseases. For example, the introduction of inhalation therapy with bronchodilators and corticosteroids for the treatment of asthma, guidelines recommending a restrictive policy towards prescribing antibiotics for respiratory symptoms [9,10], vaccination programmes against respiratory diseases (e.g. influenza and *Streptococcus pneumoniae*), and demographic changes such as population growth and the influx of ethnic minorities [11,12]. Therefore, this study investigated morbidity and hospital admission patterns for respiratory diseases in children at the turn of this century. Linking the data of a large national survey of general practice with admission data from the national medical registration enabled us to determine hospital admission rates and risk factors for hospital admission for children aged 0-17 years who presented with various respiratory diseases in general practice.

## Methods

Data were derived from the most recent nationwide study, the Second Dutch National Survey of General Practice (DNSGP-2) conducted in 2001 by the Netherlands Institute for Health Services Research (NIVEL) [13]. The Dutch National Medical Registration, maintained by the Dutch Centre for Health Care Information, was asked to provide data on all admissions of all children included in the DNSGP-2 database.

### Second Dutch National Survey of General Practice (DNSGP-2)

During the one-year registration period (i.e. 2001), 195 general practitioners (GPs) in 104 practices throughout the Netherlands participated in data collection. In the Netherlands, general practices have a fixed patient list, all inhabitants are listed in a general practice, and GPs have a gate-keeping role for specialized care. The patients enlisted in the participating practices were comparable to the general Dutch population with respect to age, gender, and type of healthcare insurance.

Data on all physician-patient contacts, prescriptions and referrals during the 12 months were extracted from the electronic medical records of all children aged 0-17 years listed in the participating practices. All diagnoses were coded using the International Classification of Primary Care (ICPC) [14]. For the current analysis, data from 14 of the 104 practices were excluded because data were incomplete due to suboptimal recording quality. Therefore, the study included 90 practices with a total of 79,272 children.

### Dutch National Medical Registration

The National Medical Registration contains information on all admissions (225,000 per annum) to all (teaching and general) hospitals in the Netherlands. Patient characteristics such as birth date, gender, postal code, and diagnostic and therapeutic interventions are registered. Admission and discharge dates, and cause of death during hospital admission are also registered. All diagnoses in the National Medical Registration are coded by trained coding clerks using the International Classification of Diseases (ICD-9) [15]. Admissions for respiratory diseases are defined according to the ICD-9 (see Additional file 1: Appendix). For the present study, we focussed on the principal diagnoses, and categorized these diagnoses into seven groups: nasopharyngitis, laryngitis and sinusitis (ICD 460-464, 472, 473, 476), acute upper respiratory infections (ICD 465), acute bronchitis and bronchiolitis (ICD 466), chronic disease of tonsil and adenoids, peritonsillar abscess (ICD 474-475), pneumonia and influenza (ICD 480-487), asthma (ICD 490-496), and other diseases of respiratory system (ICD 470, 471, 477, 478, 488, 507, 510-519). In case of multiple admissions we only included the first admission during the registration period.

### Linking National Medical Registration to DNSGP-2

Patients in both databases were linked through the combination of date of birth, sex and the four-digit postal code [16].

**Figure 1:**
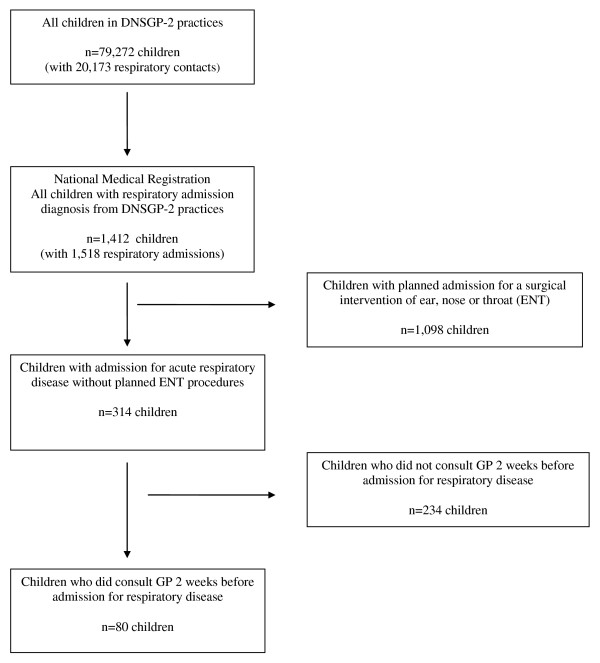
**Selection of the study population (aged 0-17 years) from the Second Dutch National Survey of General Practice (DNSGP-2) matched with hospital admissions for respiratory illness (Dutch National Medical Registration)**.

The DNSGP-2 included 79,272 children aged 0-17 years. During the 2001 registration period, for these children a diagnosis of a respiratory disease was registered 20,173 times by the GPs during a first contact of an episode. During the same period, the National Medical Registration contained 1,518 admissions for all respiratory diagnoses for the 1,412 children in the DNSGP-2. Figure 1 shows the selection process for the study population.

For the identification of determinants predicting hospital admission for an acute respiratory illness, we assumed that hospital admissions for ear, nose or throat (ENT) problems (ICD 474 and 475) were planned hospital admissions for surgical interventions. These planned ENT admissions were excluded.

### Characteristics of patients and GPs

The following characteristics were derived from the computerized patient files of DNSGP-2: age and sex of the child; season in which the GP consultation occurred (October-March versus April-September); severity of the respiratory complaint or disease [severe (R02 Shortness of breath/dyspnoea; R76 Tonsil abscess; R77 Laryngitis/tracheitis acute; R78 Acute bronchitis/bronchiolitis; R81 Pneumonia; R82 Pleurisy/pleural effusion; R96 Asthma) versus not-severe (all other diagnoses)]; single-handed practice, or not. The degree of urbanisation of the patients' living area was derived from the general practice postal code and categorized into four classes: 'less than 30,000 inhabitants', '30,000-50,000 inhabitants', 'more than 50,000 inhabitants', and 'the three largest Dutch cities Amsterdam, Rotterdam and The Hague'.

### Statistical analysis

Data on all hospital admissions for respiratory diagnoses in children aged 0-17 years were analysed. Admission rates for respiratory diseases per 1,000 children were calculated using the population size in the denominator and the number of admissions as numerator. Differences in admission rates were tested with Chi-square tests (significance level p = 0.05). To model the probability to be admitted, after a GP contact within two weeks before admission, a logistic regression model with a random practice effect was estimated and multivariate odds ratios (OR) were calculated. It was not possible to include a random effect for an individual besides the practice-specific effect. However, fitting slightly simplified models showed that the omission of this determinant had only a negligible effect on the ORs and their confidence intervals.

## Results

### General characteristics

During the one-year registration period, of the 79,272 children aged 0-17 years 1,412 (1.8%) were admitted to hospital for any respiratory diagnosis. Of these 1,412 children, 54.4% was male. Overall, 63.5% was aged 0-4 years, 24.8% 5-9 years, and 11.7% aged 10-17 years.

In total, 11% of the GP consultations were registered outside normal office hours (between 8 AM and 17 PM) or in the weekend.

Of all admitted children, 38.2% lived in an urban community of ≤ 30,000 inhabitants, 19.2% in a community of 30,000-50,000, 35% in a community of ≥ 50,000 inhabitants (excluding the 3 largest Dutch cities), and 7.6% lived in the three largest cities.

During the registration period, 94% of the 1,412 children were admitted once for a respiratory diagnosis, 61 children were admitted two times, 14 children three times, 3 children four times, and 2 children were admitted five times (a total of 1,518 admissions).

### Distribution of children and admissions by respiratory diagnosis group

Table 1 shows that the majority of children (73%) were admitted with a chronic disease of tonsils, adenoids and/or peritonsillar abscesses. Of the remaining six respiratory diagnosis groups, each group accounted for about 5% of all admissions. Among these, pneumonia and influenza (5.7%) and asthma (6.7%) were the most frequent reasons for admission.

**Table 1:** Number of admissions and duration of hospital stay per diagnosis for children aged 0-17 years.

ICD codes*	Diagnoses	No. of children (%)	No. of admissions (%)	Duration of hospital stay in%		
				**≤24 hours**	**2-7 days**	**≥8 days**
460-464, 472, 473, 476	(Acute + chronic) nasopharyngitis, laryngitis, tracheitis, sinusitis	62 (4.1)	66 (4.2)	48	50	2
465	Acute upper respiratory infections	58 (3.9)	60 (4.0)	10	74	16
466	Acute bronchitis and bronchiolitis	50 (3.3)	51 (3.4)	0	70	30
474-475	Chronic disease of tonsils and adenoids, peritonsillar abscess	1098 (73.2)	1075 (70.8)	87	12	1
480-487	Pneumonia and influenza	80 (5.3)	87 (5.7)	3	71	26
490-496	Asthma	77 (5.2)	101 (6.7)	7	70	23
470, 471, 477, 478, 488, 507, 510-519	Other diseases of respiratory system	75 (5.0)	78 (5.2)	31	57	12

**Total**		1500 (100) ^#^	1518 (100)	26	58	16

* see Additional file 1: Appendix

^# ^total number of children is higher than 1,412 because some children had more than one diagnosis

The diagnostic categories 'chronic diseases of tonsils, adenoids and peritonsillar abscess', 'nasopharyngitis, laryngitis and sinusitis', and the remainder group 'other respiratory diagnoses' had the most admissions and discharges within 24 hours (87%, 48% and 31%, respectively). Most children with 'acute upper respiratory infections', 'pneumonia and influenza', 'acute bronchitis and bronchiolitis', and 'asthma' had a hospital stay of 1-7 days (74%, 70%, 71% and 70%, respectively). A longer hospital stay of 8-30 days was seen in children with 'acute bronchitis and bronchiolitis', 'pneumonia and influenza' and 'asthma' (30%, 26%, 23%, respectively).

### Admission rates by diagnosis

The highest admission rates per 1000 children were for 'chronic disease of tonsils and adenoids', 'pneumonia and influenza', and 'asthma' (12.9, 0.97, and 0.92, respectively) (Table 2). Boys were admitted more frequently than girls. In all groups, admission rates were significantly higher among children aged 0-4 years (39/1000) than in older age groups (5-9 years: 16/1000; 10-17 years: 4.8/1000).

**Table 2:** Admission rates for respiratory diseases per 1000 children.

	All	Gender		Age					Urbanisation				
**Diseases (ICD codes)**		**Male**	**Female**	**p-value**	**0-4 years**	**5-9 years**	**10-17 years**	**p-value**	**< 30,000**	**30,000 - 50,000**	**> 50,000 excl. the 3 largest cities**	**3 largest cities**	**p-value**

Nasopharyngitis, laryngitis, sinusitis (460-464, 472, 473, 476)	0.75	0.71	0.80	0.64	1.33	0.44	0.56	< 0.01	0.68	0.96	0.78	0.52	0.66
Acute upper respiratory infections (465)	0.71	0.97	0.43	< 0.01	2.17	0.13	0.08	< 0.01	0.59	0.32	0.93	1.38	0.02
Acute bronchitis, bronchiolitis (466)	0.61	0.59	0.63	0.85	2.00	0.09	0	< 0.01	0.47	0.57	0.71	1.04	0.34
Chronic disease of tonsil and adenoids, peritonsillar abscess (474-475)	12.86	12.96	12.75	0.79	26.90	13.84	2.75	< 0.01	12.55	13.13	13.34	11.74	0.70
Pneumonia and influenza (480-487)	0.97	1.28	0.65	< 0.01	2.79	0.22	0.22	< 0.01	0.71	0.57	1.46	1.38	0.01
Asthma (490-496)	0.92	1.28	0.55	< 0.01	2.46	0.44	0.20	< 0.01	0.47	1.27	1.12	1.73	< 0.01
Other diseases of respiratory system (470, 471, 477, 478, 488, 507, 510-519)	0.90	1.06	0.73	0.10	1.29	0.40	0.96	< 0.01	0.82	0.57	1.12	1.21	0.24
TOTAL	17.72	18.85	16.54	0.03	38.94	15.56	4.77	< 0.01	16.29	17.39	19.46	19.00	0.08

Children with 'acute upper respiratory infections', 'pneumonia and influenza' and 'asthma' were significantly more frequently admitted to hospital when living in a more urbanised region. In total, 3,417 children in the DNSGP-2 database consulted their GP for asthma and 77 (2.3%) of these children were admitted to hospital. The admission rate for asthma in rural areas was 0.47 per 1000 children per year; in suburban areas it was 1.27; in urban areas it was 1.12; and for the 3 largest cities the admission rate was 1.73 (p = 0.002).

Of all children admitted with a respiratory diagnosis, one died in hospital. This was an 8-month-old child admitted with pneumonia (ICD 486) who had a severe underlying neuromuscular disease.

Of the 1,412 children, 323 (23%) children had 396 admissions for an ICD diagnosis that we considered not to be planned (Figure 1).

### Distribution of diagnoses and GP consultation

Of the 80 children who consulted their GP two weeks before hospital admission, a higher percentage of children were admitted with 'nasopharyngitis, laryngitis, tracheitis, sinusitis', 'bronchitis and bronchiolitis' and 'pneumonia and influenza' compared with children who did not consult the GP (Table 3). In the group of 234 children who did not consult the GP two weeks before hospital admission, two differences showed significance. A lower percentage of children in this group were admitted with acute bronchitis and bronchiolitis (p = 0.02) and a higher percentage of children were admitted with asthma (p < 0.001).

**Table 3:** Distribution of diagnoses for all children with admission for acute respiratory disease without ENT procedures (n = 314), for children who did consult GP two weeks before admission for respiratory disease (n = 80) and for children who did not consult GP two weeks before admission for respiratory disease (n = 234).

ICD codes	Diagnoses	All children (%) (n = 314)	Children who consulted GP (%) (n = 80)	Children who did not consult GP (%) (n = 234)
460-464, 472, 473, 476	(Acute + chronic) nasopharyngitis, laryngitis, tracheitis, sinusitis	29 (9.2)	10 (12.5)	19 (8.1)
465	Acute upper respiratory infections	55 (17.5)	15 (18.8)	40 (17.1)
466	Acute bronchitis and bronchiolitis	49 (15.6)	19 (23.8)	30 (12.8)
480-487	Pneumonia and influenza	78 (24.8)	23 (28.7)	55 (23.5)
490-496	Asthma	73 (23.3)	6 (7.5)	67 (28.6)
470, 471, 477, 478, 488, 507, 510-519	Other diseases of respiratory system	30 (9.6)	7 (8.7)	23 (9.7)

### Determinants predicting hospital admission for respiratory diseases

Table 4 shows the adjusted ORs obtained from the logistic regression model. The multivariate logistic regression showed that younger age and more severe respiratory diseases predict a hospital admission in the two weeks after GP consultation. None of the other included determinants had an independent effect on hospital admission.

**Table 4:** Relation between child and GP determinants and hospital admission for respiratory disease (n = 95 GP contacts two weeks before admission).

	Relative probability of hospital admission within 2 weeks after GP consultation (n = 95) OR (95% CI)	p-value
Age of child (years)	0.81 (0.76-0.88)	< 0.001
Sex:		
Boy	1.14 (0.63-1.66)	0.55
Girl	(ref)	
Season:		
October-March	1.18 (0.64-1.73)	0.56
April-September	(ref)	
Severity:		
severe	5.55 (2.99-8.11)	< 0.001
not severe	(ref)	
Urbanisation:		
rural	0.96 (0.40-1.52)	0.89
suburban	0.92 (0.20-1.64)	0.84
urban	(ref)	
Practice:		
not single-handed	0.75 (0.34-1.16)	0.30
single-handed	(ref)	

Multivariate logistic regression analysis.

## Discussion

In this nationally representative study population, admission to hospital for a respiratory diagnosis occurred in only 1.8% of children. Children aged < 4 years with respiratory diagnoses were admitted more often. Admission rates for acute respiratory tract infections, pneumonia and influenza, and asthma were higher in urban regions than in rural areas. Admissions for asthma were not common, i.e. about 0.1% of all children, and 2.3% of all children who consulted their GP with asthma. Many of the admissions were planned (77%), mostly for ENT procedures. Children with upper respiratory infections, pharyngitis, sinusitis, bronchitis and pneumonia consulted their GP relatively more often in the two weeks before admission. In contrast, children with asthma and other diseases consulted their GP less often before admission. Younger age and a more severe respiratory disease predicted hospital admission in the two weeks after the GP consultation.

The major strength of this study is the use of unique data from a large national survey and the linking of these data to the Dutch National Medical Registration. The study population allowed to calculate admission rates for all respiratory diseases. Characteristics of the study patients are comparable to the general Dutch population, and the GP sample is comparable to the national GP population [17]. Single-handed practices were somewhat underrepresented; however, because this item is not related to study outcome it is not considered an important limitation. The results can be assumed to represent regular primary care and consultation behaviour in the Netherlands.

About 11% of the GP consultations were registered outside normal office hours or in the weekend. Although this may entail some underrepresentation, we don't believe this limits generalizability.

We could not test the assumption that all admissions for ICD codes 474 and 475 were planned. However, over 99% of these admissions were for ICD code 474, which concerns chronic disease of tonsils and adenoids. In the Netherlands, the vast majority of admissions under this diagnosis is due to planned (adeno)tonsillectomy.

2.3% of all children who consulted their GP for asthma were admitted to hospital. The denominator of this proportion underestimates the true number of asthmatic children since not all asthmatic children (mainly children suffering from intermittent asthma) may have consulted their GP within the study period.

Although the data we used are several years old, we do not believe this is a major limitation. The outcome (low admission rates) would not differ greatly when more recent data would have been available. We used the most recent nationwide study for answering our research question.

The present study provides insight into the large differences in disease profiles for children treated in general practice or in hospital. For example, in general practice 5% of the presented respiratory problems concern pneumonia [18]. In contrast, 25% of the children with a respiratory problem were admitted to hospital with pneumonia (Table 3). On the other hand, 17% of children admitted to hospital suffered from acute upper respiratory infections compared with 32% of the children in general practice. The relative contribution of asthma was about 22% in both general practice and in hospital [18]. The considerable difference in disease presentation of children consulting GPs or hospital physicians was reported by Hodgkin in his classic work dating from 1978 [19].

Of the 314 children with an unplanned admission to hospital, only 25% attended general practice with respiratory complaints two weeks before admission. Of the 234 (75%) children who did not consult the GP two weeks before admission, 45% did not visit their general practice at any time during the one-year registration period. We assume that these children were already receiving specialised care.

Children with acute upper respiratory infections, pneumonia, influenza and asthma were admitted to a hospital significantly more often when living in an urbanised region. This might be due to greater air pollution in urban regions. The impact of air pollution on respiratory admissions and asthma has been reported in Canada and Italy [20,21]. Another explanation could be the availability of hospitals in urban regions.

Gender and age differences are common in hospitalization rates for respiratory diseases [22,23]. In the present study we also found that younger children and boys were admitted more often than older children and girls.

The finding that children with more severe illness are more likely to get admitted, is not surprising. More surprising is that no other determinants turned out to be related to admission.

The present study found very low admission rates for respiratory diseases, especially for asthma. Hospital admission is often used as quality parameter [24,25]. The low admission rates in our study show that it may be necessary to reconsider the use of hospital admission as a quality parameter for asthma treatment, and that for effectiveness studies in primary care settings admission rates should no longer be used as a quality end-point.

## Conclusions

Children in the general population with respiratory complaints/diseases had very low hospital admission rates. There is a large difference in the scope of work of GPs and specialists in relation to respiratory diseases in children. In urban regions children were more frequently admitted with upper respiratory infections, pneumonia, influenza and asthma. Finally, we suggest that for effectiveness studies in a primary care setting the hospital admission rate should not be used as quality end-point.

## Competing interests

The authors declare that they have no competing interests.

## Authors' contributions

JHJMU participated in the study design, was involved in the statistical analysis, and drafted the manuscript. FGS participated in the study design and revised the manuscript critically. PJEB participated in the study design and revised the manuscript critically.

SPW performed statistical analyses and helped to draft the manuscript. JCvdW participated in the study design, was involved in the statistical analysis, and revised the manuscript critically. All authors read and approved the final manuscript.

## Authors' information

JHJMU: MD, GP., FGS: MD, PhD, professor of general practice., PJEB: MD, PhD, professor of general practice., SPW: statistician., JCvdW: PhD, senior lecturer.

## Pre-publication history

The pre-publication history for this paper can be accessed here:

http://www.biomedcentral.com/1471-2296/11/76/prepub

## Supplementary Material

Additional file 1 **Appendix ICD-9 codes**. ICD-9 codes used to select all children admitted with a respiratory diagnosis. Click here for file
