# Supplementary material for: Low hospital admission rates for respiratory diseases in children
Source: BMC Fam Pract. 2010 Oct 9;11:76. doi: 10.1186/1471-2296-11-76 (PMC2958964; doi:10.1186/1471-2296-11-76)
Supplement: Additional file 1 — Appendix ICD-9 codes. ICD-9 codes used to select all children admitted with a respiratory diagnosis. [file 1471-2296-11-76-S1.DOC]

**ICD-9 codes used to select all children admitted with a respiratory diagnosis.**

460 acute nasopharyngitis

461 acute sinusitis

462 acute pharyngitis

463 acute tonsillitis

464 acute laryngitis and tracheitis

465 acute upper respiratory infections of multiple or unspecified sites

466 acute bronchitis and bronchiolitis

470 deviated nasal septum

471 nasal polyps

472 chronic pharyngitis and nasopharyngitis

473 chronic sinusitis

474 chronic disease of tonsils and adenoids

475 peritonsillar abscess

476 chronic laryngitis and laryngotracheitis

477 allergic rhinitis

478 other diseases of respiratory tract

480 viral pneumonia

481 pneumococcal pneumonia

482 other bacterial pneumonia

483 pneumonia due to other specified organism

484 pneumonia in infections diseases classified elsewhere

485 bronchopneumonia, organism unspecified

486 pneumonia, organism unspecified

487 influenza

488 influenza due to identified avian influenza virus

490 bronchitis, not specified as acute or chronic

491 chronic bronchitis

492 emphysema

493 asthma

494 bronchiectasis

495 extrinsic allergic alveolitis

496 chronic airway obstruction, not elsewhere classified

507 pneumonitis due to solids and liquids

510 empyema

511 pleurisy

512 pneumothorax

513 abscess of lung and mediastinum

514 pulmonary congestion and hypostasis

515 post inflammatory pulmonary fibrosis

516 other alveolar and parietoalveolar pneumopathy

517 lung involvement in conditions classified elsewhere

518 other diseases of lung

519 other diseases of respiratory system
